# Cortical unlike trabecular bone loss is not associated with vascular calcification progression in CKD patients

**DOI:** 10.1186/s12882-020-01756-2

**Published:** 2020-04-06

**Authors:** Larissa R. Costa, Aluizio B. Carvalho, Amandha L. Bittencourt, Carlos E. Rochitte, Maria Eugênia F. Canziani

**Affiliations:** 1 Nephrology Division of Federal University of São Paulo, Rua Pedro de Toledo, 282 - Vila Clementino, São Paulo, SP 04039-000 Brazil; 2 Heart Institute of the University of São Paulo, Av. Dr. Enéas Carvalho de Aguiar, 44 - Pacaembu, São Paulo, SP 05403-900 Brazil

**Keywords:** Cortical bone, Trabecular bone, Vascular calcification, Chronic kidney disease

## Abstract

**Background:**

Vascular calcification progression has been associated with the loss of trabecular bone in chronic kidney disease (CKD) patients. There are few data evaluating the relationship between cortical bone loss and vascular calcification in this population. The aim of this study was to prospectively evaluate the association between changes in cortical bone density and coronary artery calcification (CAC) progression in non-dialyzed CKD patients.

**Methods:**

Changes of cortical and trabecular bone, and changes of calcium score, were analyzed using vertebral tomographic images from a prospective study. Automatic delineation of the cortical bone layer was performed by *Image J* software, and trabecular bone was determined by selecting a region of interest using Vitrea 2® software. Cortical and trabecular bone density (BD) were expressed in Hounsfield Units (HU), and coronary artery calcium score in Agatston Units (AU).

**Results:**

Seventy asymptomatic patients [57.8 ± 10.2 years, 63% males, 20% diabetic, estimated glomerular filtration rate (eGFR) = 37.3 (24.8–51.3) mL/min/1.73m^2^] were followed for 24 months. The mean cortical and trabecular BD did not change over time. While 49 patients lost either bone, 29 (41%) patients lost cortical [− 4.4%/year (ranging from − 7.15 to − 0.5)] and 39 (56%) lost trabecular bone [− 3.15%/year (− 13.7 to − 0.25)]. There was no association between cortical and trabecular BD changes (*p* = 0.12). CAC was observed in 33 (46%) patients at baseline, and 30 (91%) of them showed CAC progression. While an inverse correlation between trabecular bone and calcium score changes was observed (*p* = 0.001), there was no correlation between cortical bone and calcium score changes (*p* = 0.34).

**Conclusion:**

CKD patients experience either cortical or trabecular bone loss over time, but these changes do not take place simultaneously in all patients. Cortical, unlike trabecular bone loss, is not associated with vascular calcification progression in these patients.

## Background

Structural and metabolic differences between trabecular and cortical bone have already been described. While cortical bone is a dense, low-porosity and less metabolically active tissue, trabecular bone is a honeycomb-like trabecular network with a larger remodeling area and higher turnover rate [1]. These intrinsic features go along with different key roles of each type of bone tissue, e.g., mechanical strength for cortical and mineral homeostasis for trabecular bone [2].

The disturbed bone remodeling cycle present on chronic kidney disease-mineral bone disorders (CKD-MBD) impairs bone turnover and mineralization, leading to reduced bone mass and quality [3]. Most of the knowledge on bone metabolism in chronic kidney disease (CKD) patients comes from studies performed on trabecular bone. However, recently, a prospective study showed an increase of cortical porosity in hemodialysis patients, suggesting that cortical bone may contribute to the bone loss observed in this population [4].

Bone loss has been associated with vascular calcification and both contribute to high morbidity and mortality rates in CKD patients [5]. Data regarding this association, using Dual-energy X-ray absorptiometry (DXA), have controversial results [6], as spine bone mineral density (BMD) may be overestimated by the presence of aorta calcification and osteoarthritis. Cross-sectional [7] and prospective [8] studies using quantitative computerized tomography (QCT) have shown that trabecular bone loss has been associated with vascular calcification in CKD patients. This method has shown to be a better alternative to DXA, as besides reducing BMD measurement errors, it allows trabecular and cortical bone differentiation [3, 9]. Moreover, Carvalho et al. [10] have shown that trabecular bone density, measured at the thoracic vertebra through QCT imaging, correlated to bone histomorphometric parameters in hemodialysis patients.

There is no study evaluating cortical bone and vascular calcification in the CKD scenario. It is important to mention that cortical bone comprises 80% of the human skeleton and one could hypothesize that any loss of bone from this compartment could represent a huge load of calcium driven to vessels. Therefore, the aim of this study was to evaluate, through QCT images, the relationship between changes of cortical bone density and coronary artery calcification (CAC) progression in non-dialyzed CKD patients.

## Methods

### Patients and study protocol

This study is a post hoc analysis of a prospective study that aimed to investigate the association of trabecular bone changes and vascular calcification progression during a 24-month follow-up [8]. In that study, 72 CKD-stage 2 to 4 asymptomatic patients, from 18 to 70 years-old, were evaluated. Exclusion criteria were clinical evidence of chronic inflammatory disease, active malignancy, human immunodeficiency virus infection, viral hepatitis or chronic use of steroids and patients referred to nephrologists less than 3 months.

The patients underwent an assessment of their clinical history, physical examination and laboratory tests. CKD duration was considered as the length between the diagnosis and the enrollment into the study. Thoracic images from a multi-slice computerized tomography scanner (Somatron Volume Zoom Siemens AG, Erlangen, Germany) were evaluated to measure cortical and trabecular vertebral bone density and coronary calcium score. Cortical images were scored by 2 observers and inter-observer variability was < 1%. The data from only one observer were used for the analyses. Cortical bone images of 2 patients were not available. Therefore 70 out of 72 patients were evaluated in the present study.

The patients were on regular use of angiotensin-converting enzyme inhibitors (83%), diuretics (71%), β-blockers (43%), calcium channel blockers (37%), sevelamer (33%), statins (29%), angiotensin receptor blockers (19%), bicarbonate (17%), calcium-based phosphate binders (6%), calcitriol (6%) and erythropoietin-alpha (3%).

This study was reviewed and approved by the Ethics Advisory Committee of the Federal University of São Paulo (no. 1113186).

### Vertebral bone density

#### Cortical bone

**Fig. 1:**
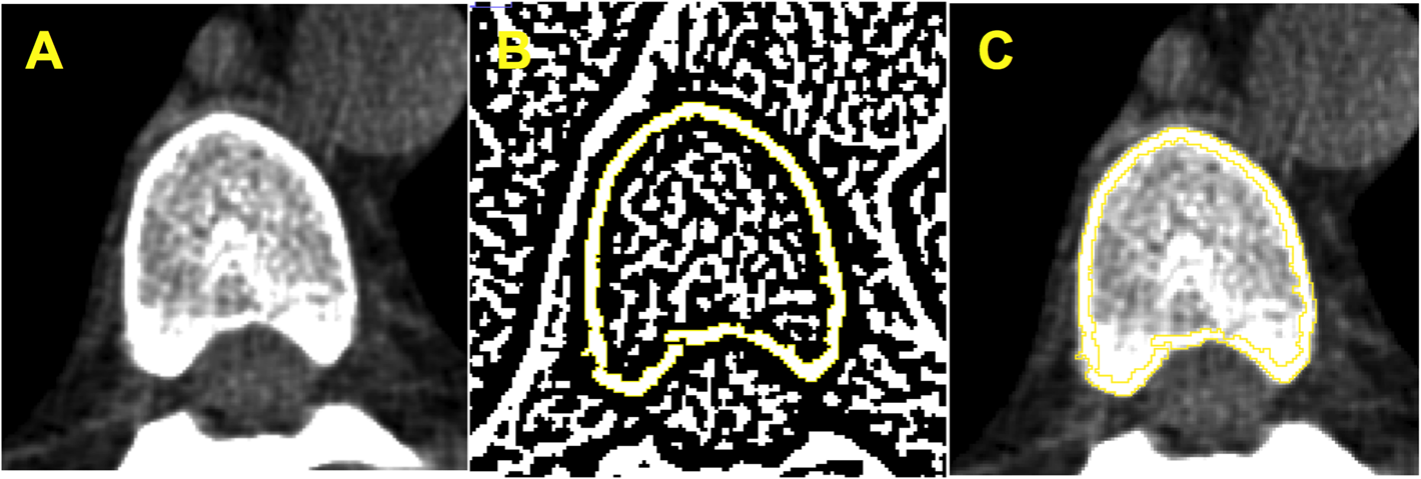
Cortical vertebral tomography. **a** Axial vertebral image selection. **b** Transformation into binary image and generation of a cortical mask through the automatic delineation of cortical bone layer performed by *Image J* software®. *C* – Overlapped cortical mask on the original image followed by automatic cortical density measurement

Cortical bone density was evaluated at baseline and 24-month, by *Image J* software (Image J 1.49v, National Institutes of Health, Bethesda, Maryland, USA, 1997–2016) [11, 12]. A DICOM image (16 bits) was selected from the vertebral body in the axial section at the level of the aortic root (Fig. 1a). This image was converted into an 8-bit image which allowed the transformation into a binary image and generation of a cortical mask through the automatic delineation of the cortical bone layer (Fig. 1b), using the threshold function with Niblack algorithm and radius 4. This generated cortical mask was overlapped on the original image (DICOM 16 bits) and cortical bone density was automatically measured (Fig. 1c). Cortical bone densities were expressed in Hounsfield Units (HU). Bone density changes were calculated as the difference between 24-month and baseline densities/baseline density*100, expressed by %/year. Bone loss was defined as any bone density change below zero.

#### Trabecular bone

**Fig. 2:**
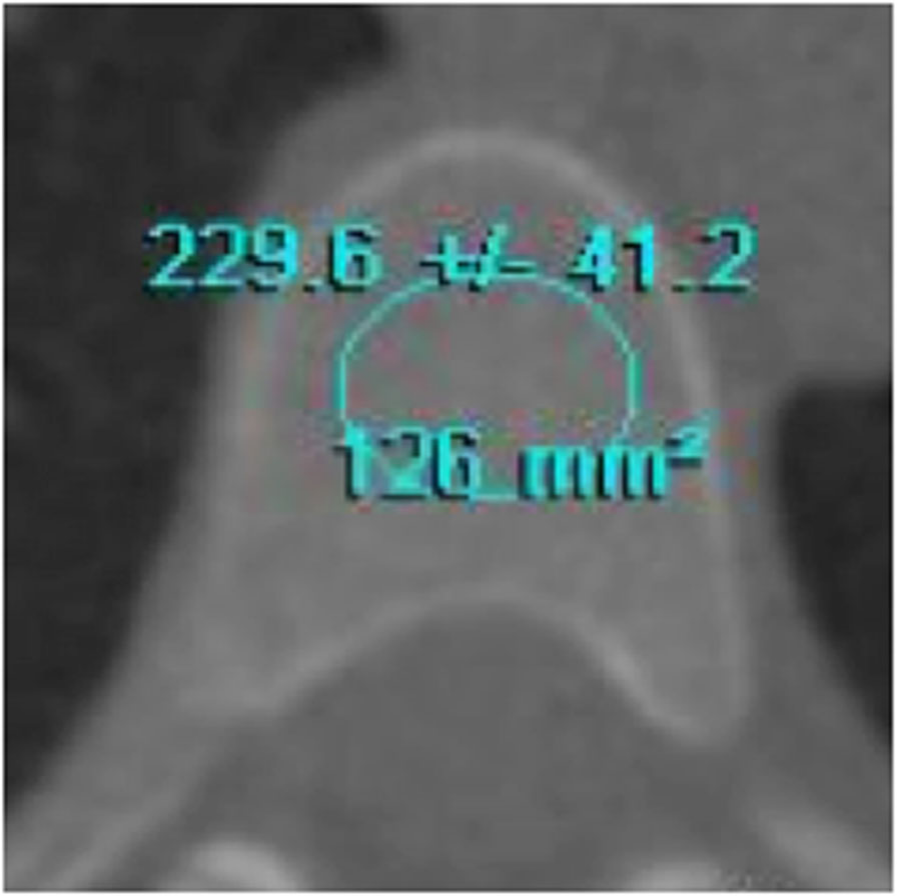
Trabecular vertebral tomography

Trabecular bone density was evaluated at baseline and 24-month by selecting a region of interest placed at mid-vertebral body (Fig. 2) using Vitrea 2® workstation software (Vital Images Inc., Plymouth, MN) [7, 10].

Trabecular bone densities were expressed in HU. Bone density changes were calculated as the difference between 24-month and baseline densities/baseline density*100, expressed by %/year. Bone loss was defined as any bone density change below zero.

### Coronary artery calcification (CAC)

The calcium score was obtained by multi-slice computerized tomography as described elsewhere [13]. Calcium score was expressed in Agatston Units (AU) and the presence of CAC was defined as calcium score ≥ 10 AU. CAC progression was calculated as the difference between 24-month and baseline scores/baseline score*100.

### Laboratory tests

Laboratory analyses at baseline and 24-month included: serum creatinine, hemoglobin, lipid profile, bicarbonate, ionized calcium, phosphate, alkaline phosphatase, 24 h proteinuria measured by standard methods, and intact parathyroid hormone (iPTH) by chemiluminescence immunoassay (Immulite; DPC-Biermann, Bad Nauheim, Germany). The glomerular filtration rate was calculated by CKD-EPI equation [14].

### Statistical analysis

All variables were presented as mean and standard deviation, median and interquartile range or frequencies. The distribution of data was evaluated by Kolmogorov-Smirnov statistical test. The continuous variables were compared using Student’s t-test or Wilcoxon, as appropriate, and proportions by McNemer tests. Univariate associations were analyzed by Pearson’s or Spearman’s tests according to the distribution of the variables.

Variables selected in univariate analyses were fed into multivariate linear regression models to verify their independent association with the change of cortical and trabecular bone.

*P*-values < 0.05 were considered statistically significant. All statistical analysis was performed using SPSS for Windows (SPSS 18.0, Chicago, IL, USA).

## Results

The baseline and 24-month demographic, laboratory and tomographic data are presented in Table 1. Patients were predominantly middle-aged males. Obesity [body mass index (BMI) ≥ 30 kg/m^2^] was found in 31% of the patients, overweight (BMI ≥ 25 kg/m^2^) in 29%, and BMI < 18.5 kg/m^2^ in only 1 patient. Diabetes and smoking were observed in 20 and 51% of the patients, respectively. According to the CKD classification [15], 8 (11%) patients were in stage 2, 14 (20%) in stage 3a, 24 (34%) in stage 3b and 24 (34%) in stage 4.

**Table 1:** Patients characteristics at baseline and 24 months (*n* = 70)

	Baseline	24 months	*p*
**Demography**			
Age (years)	57.8 ± 10.2		
Males (%)	44 (63)		
Body mass index (kg/m^2^)	26.4 (22.8–31.3)		
Diabetes (%)	14 (20)		
Smoking (%)	36 (51.4)		
CKD duration (months)	25.4 ± 1.3		
**Laboratory**			
eGFR (mL/min/1.73m^2^)	37.3 (24.8–51.3)	31.2 (20.3–51)	< 0.001
Proteinuria (g/24 h)	0.19 (0–0.49)	0.43 (0–1)	< 0.001
Hemoglobin (g/dL)	13.3 ± 1.6	12.9 ± 4.4	< 0.001
Total cholesterol (mg/dL)	183 (161–205)	158 (131–188)	< 0.001
HDL-cholesterol (mg/dL)	49 (41–57)	41 (35–48)	< 0.001
LDL-cholesterol (mg/dL)	104 (85–119)	87 (68–106)	0.001
Triglycerides (mg/dL)	129 (99–211)	127 (89–177)	0.20
Bicarbonate (mmol/L)	21.9 ± 3.0	25.5 ± 3.8	< 0.001
Phosphate (mg/dL)	3.7 (3.1–4.0)	3.4 (3.1–3.8)	0.04
Ionized calcium (mmol/L)	1.29 ± 0.06	1.32 ± 0.07	0.003
Alkaline Phosphatase (U/L)	79 (63–93)	70.5 (60.8–94)	0.17
Intact PTH (pg/mL)	88 (61–153)	98 (56.5–171)	0.63
**Tomography**			
Trabecular Bone Density (HU)	202.9 ± 50.5	202.5 ± 51	0.91
Cortical Bone Density (HU)	400 ± 89.9	404 ± 94.8	0.40
Coronary calcium score (AU)	5.5 (0–283.8)	7.5 (0–385.3)	< 0.001

*eGFR* Estimated glomerular filtration rate

During the follow-up, there was a decline in renal function and an increase in proteinuria. Total, LDL and HDL-cholesterol decreased, while triglycerides levels remained unchanged. Alkaline phosphatase and iPTH did not change over time, while ionized calcium increased and phosphate levels decreased.

**Fig. 3:**
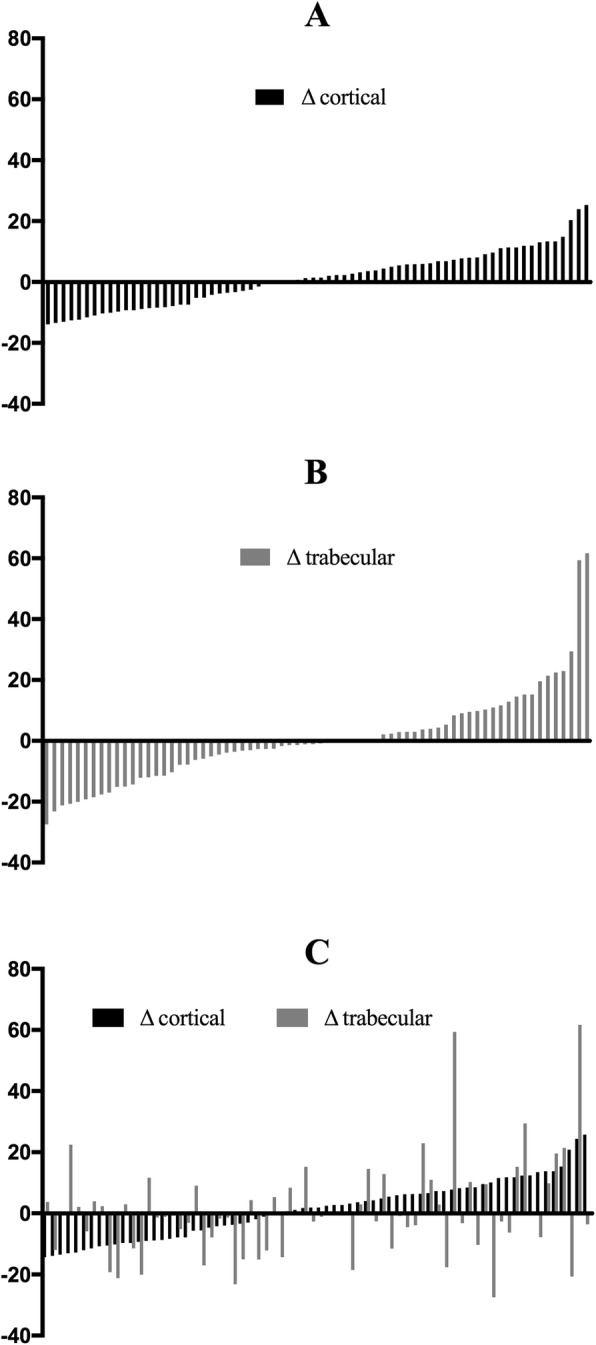
Cortical (**a**) and Trabecular (**b**) changes in bone density during the study. Cortical and Trabecular bone density (**c**) changes of each patient during the study

The mean cortical and trabecular bone density did not change. However, 49 out of 70 patients (70%) lost either cortical or trabecular bone. Regarding that, 29 (41%) patients lost cortical [− 4.4%/year (ranging from − 7.15 to − 0.5); (Fig. 3a)], while 39 (56%) lost trabecular bone [− 3.15%/year (− 13.7 to − 0.25); (Fig. 3b)], over time. Figure 3c shows the changes in the cortical and trabecular bone of each patient. Nineteen (27%) patients lost cortical and trabecular bone simultaneously.

Coronary calcium scores significantly increased during the study (Table 1). CAC was observed in 33 (46%) patients at baseline and CAC progression in 30 (91%) out of them.

Table 2 depicts the correlations between cortical and trabecular bone density changes with the other variables. Cortical bone change correlated to HDL-cholesterol (r = 0.26; *p* = 0.03), while trabecular bone change inversely correlated to the duration of CKD (r = − 0.25; *p* = 0.04). The cortical bone change was associated with smoking (− 1.07 ± 8.2 vs. 3.73 ± 10.5 HU, smoker and non-smoker, respectively; p = 0.04). No difference in cortical and trabecular bone changes regarding gender, presence of diabetes, obesity, use of medicines or other laboratory parameters was observed. Noticeable, no correlation between cortical and trabecular bone density changes (r = 0.19; *p* = 0.12) was observed.

**Table 2:** Univariate analysis of cortical and trabecular vertebral bone density changes

	Cortical Change		Trabecular Change	
	*r*	*p*	*r*	*p*
Age	0.01	0.96	0.12	0.31
CKD duration	−0.04	0.74	−0.25	0.04
Body mass index	0.11	0.35	0.12	0.33
Δ eGFR	0.02	0.90	0.22	0.07
Δ LDL-cholesterol	0.09	0.46	−0.15	0.24
Δ HDL-cholesterol	0.26	0.03	0.05	0.69
Δ triglycerides	−0.02	0.86	−0.02	0.85
Δ bicarbonate	0.11	0.37	0.25	0.05
Δ phosphorus	−0.02	0.90	0.02	0.91
Δ ionized calcium	−0.05	0.68	0.55	0.66
Δ alkaline phosphatase	−0.29	0.82	0.83	0.50
Δ intact PTH	−0.04	0.75	−0.08	0.51
Δ trabecular bone density	0.19	0.12	–	–
Δ cortical bone density	–	–	0.19	0.12

Δ = change, *CKD* Chronic kidney disease, *eGFR* Estimated glomerular filtration rate

**Fig. 4:**
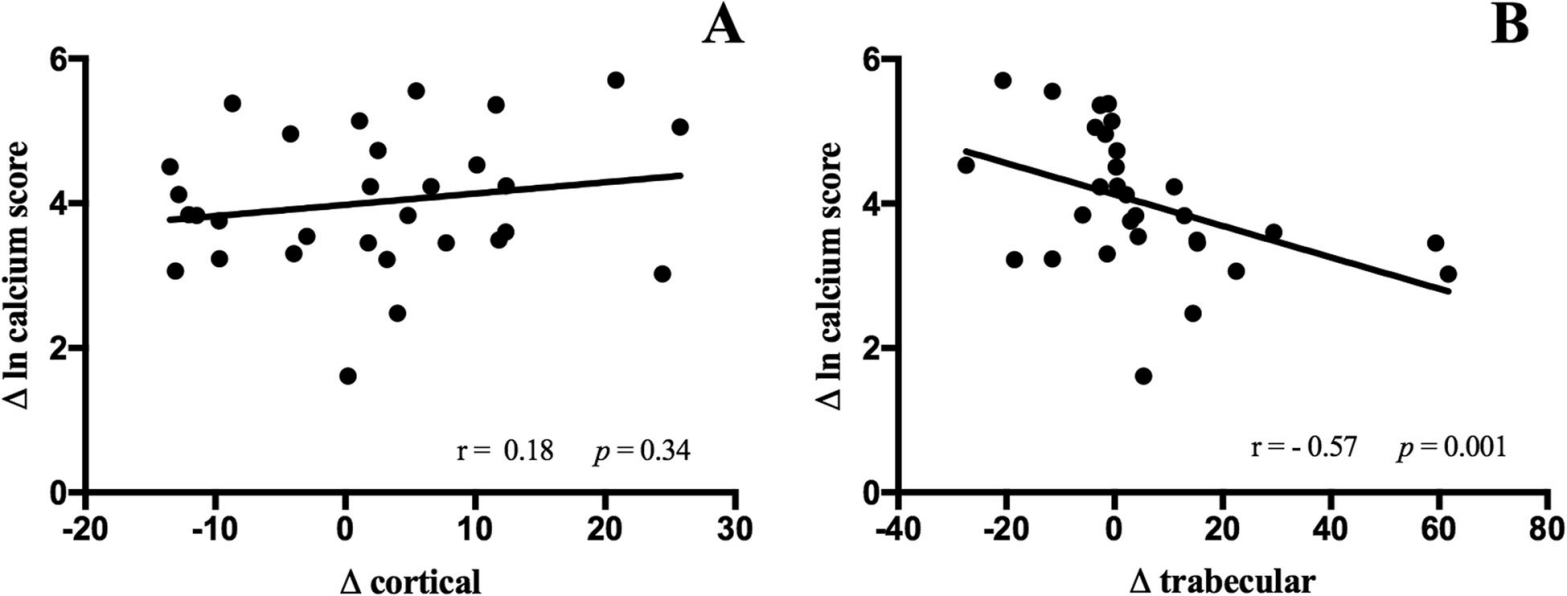
Correlation between calcium score change and cortical (**a**) and trabecular (**b**) bone density changes

Figure 4 shows the relationship between cortical (Fig. 4a) and trabecular (Fig. 4b) bone densities with vascular calcification progression. There was no correlation between cortical bone and calcium score changes, while an inverse correlation between changes of trabecular bone and calcium score was observed.

Multivariate models analysis showed no correlations between cortical changes and calcium score changes, even after adjusted to HDL changes and smoking (*p* = 0.11; IC 95% -0.6 / 5.5). Trabecular changes correlated to calcium score changes, adjusted to duration of CKD (*p* = 0.03; IC 95% 3.3 / -0.2).

There was no difference in calcium score change between patients who have lost or gained cortical bone (92.1 ± 87 vs. 68.7 ± 60.6 AU, respectively; *p* = 0.32). On the other hand, patients who have lost trabecular bone had a higher calcium score change compared to those who gained (134 ± 94 vs. 45 ± 28 AU, respectively; *p* = 0.005).

## Discussion

This is a prospective study that assessed cortical and trabecular bone density changes and their relationship with vascular calcification progression in CKD patients. During the study, about half of the patients lost either cortical or trabecular bone. It is worth mentioning that both losses did not take place simultaneously in all patients. In addition, vascular calcification progression occurred in almost all calcified patients and was not associated, in the same manner, to cortical or trabecular bone loss.

The link between bone loss and cardiovascular disease either in CKD [16–19] or non-CKD population [20] has been extensively discussed in the literature. In the present study, using vertebral QCT images, we could show a distinct potential role of each type of bone tissue on vascular calcification progression. Confirming previous data, there was an association between CAC progression and trabecular bone loss [8, 21]. To the best of our knowledge there was no study evaluating cortical bone and vascular calcification in CKD scenario. In the present study, no relation between cortical bone loss and vascular calcification was observed. This opposite behavior, between cortical and trabecular bone, could be explained by different structural and metabolic characteristics of either bone tissues.

Compared to trabecular bone, cortical bone is denser, has lower porosity and turnover rate. These characteristics grant to cortical bone a relevant biomechanical strength function. On the other hand, the trabecular bone has a larger remodeling area and a higher turnover rate indicating an active and relevant metabolic function [1, 2]. Thus, the lower metabolic cortical activity, may explain the lack of association between cortical bone loss and vascular calcification progression observed in this study.

It is well known that bone loss, disregarding the method of measurement, is a frequent finding in CKD, with a prevalence varying up to 100% of the patients [22, 23]. Similarly, in the present study, 70% of the patients experienced cortical and/or trabecular bone loss after 2 years of follow-up. On the other hand, data concerning the magnitude of bone loss varies widely. In the present study a high rate of bone loss was observed (− 4.4%/year for cortical and − 3.1%/year for trabecular bone). Previous prospective studies in CKD patients, found a rate of bone loss, ranging from − 4.1 to − 0.7%/year, depending on the analyzing method (DXA or QCT), the skeletal site (hip or radius), the population characteristics (predialysis or dialysis) and the presence of fracture [23, 24].

Among those factors, the image method employed seems to be one of the utmost importance. In fact, DXA, the most common and available method used, is an imaging test that measures bone mass at specific sites. This method cannot discriminate trabecular and cortical bone, and may overestimate bone density measurements due to the presence of some conditions, such as arthritis and vascular calcification [25–27]. On the other hand, high-resolution imaging tools, as high-resolution peripheral QCT (HD-pQCT), are able to evaluate bone density and microarchitecture, as well. However, this is a high-cost and routinely worldwide unavailable method [28–31]. Finally, thoracic vertebral QCT showed to be a feasible tool. This new method, employed in the present study, allows simultaneous and prospective assessment of vascular calcification and bone mass changes over time. Moreover, it offers the possibility of a non-invasive and more cost-effective method that evaluates separately cortical and trabecular bone density without contamination of osteophytes and aortic calcification [32, 33].

Limitations of this study should be addressed. It is a post hoc study, with a relative small sample size that did not permit to calculate the power of such study. Moreover, the method of cortical bone measurement has not been validated yet, and its least significant change was not established. However, the scientific image-analysis program, Image J, has been widely used in the health field [11, 12] and recently, the evaluation of the cortical bone by image methods has been considered more accurate than the gold-standard method, e.g., bone biopsy [34, 35]. Noteworthy, we have previously validated the use of QCT for trabecular bone in predialysis CKD patients [10]. Moreover, other authors using HR-pQCT could demonstrate an inverse correlation between cortical density and cortical bone porosity, in hemodialysis patients [36].

## Conclusion

CKD patients experience either cortical or trabecular bone loss over time, but these changes do not take place simultaneously in all patients. Cortical, unlike trabecular bone loss, is not associated with vascular calcification progression in these patients.

## Data Availability

The datasets used and/or analyzed during the current study are available from the corresponding author on reasonable request.

## Abbreviations

CKD-MBD: Chronic kidney disease-mineral bone disorders
CKD: Chronic kidney disease
DXA: Dual-energy X-ray absorptiometry
BMD: Bone mineral densitometry
QCT: Quantitative computed tomography
CAC: Coronary artery calcification
HU: Hounsfield Unit
AU: Agatston Unit
iPTH: Intact parathyroid hormone
CKD-EPI: Chronic kidney disease epidemiology collaboration
BMI: Body mass index
LDL: Low-density lipoprotein
HDL: High-density lipoprotein
HR-pQCT: High resolution peripheral quantitative computerized tomography
